# Inequity in maternal health care utilization in Vietnam

**DOI:** 10.1186/1475-9276-11-24

**Published:** 2012-05-15

**Authors:** Emilia Goland, Dinh Thi Phuong Hoa, Mats Målqvist

**Affiliations:** 1 International Maternal and Child Health, Department of Women’s and Children’s Health, Uppsala University, Uppsala, Sweden; 2 Hanoi School of Public Health, Hanoi, Vietnam

**Keywords:** Maternal health, Health care utilization, Equity, Inequity, Antenatal care, Skilled birth attendance, Ethnicity, Social determinants of health, Vietnam

## Abstract

**Introduction:**

Vietnam has succeeded in reducing maternal mortality in the last decades. Analysis of survey data however indicate that large inequities exist between different segments of the population. We have analyzed utilization of antenatal care and skilled birth attendance among Vietnamese women of reproductive age in relation to social determinants with the aim to reveal health inequities and identify disadvantaged groups.

**Method:**

Data on maternal health care utilization and social determinants were derived from the Multiple Indicator Cluster Survey (MICS) conducted in Vietnam in 2006, and analyzed through stratified logistic regressions and g-computation.

**Results:**

Inequities in maternal health care utilization persist in Vietnam. Ethnicity, household wealth and education were all significantly associated with antenatal care coverage and skilled birth attendance, individually and in synergy. Although the structural determinants included in this study were closely related to each other, analysis revealed a significant effect of ethnicity over and above wealth and education. Within the group of mothers from poor households ethnic minority mothers were at a three-fold risk of not attending any antenatal care (OR 3.06, 95% CI 1.27–7.41) and six times more likely not to deliver with skilled birth attendance (OR 6.27, 95% CI 2.37–16.6). The association between ethnicity and lack of antenatal care and skilled birth attendance was even stronger within the non-poor group.

**Conclusions:**

In spite of policies to out rule health inequities, ethnic minority women constitute a disadvantaged group in Vietnam. More efficient ways to target disadvantaged groups, taking synergy effects between multiple social determinants into consideration, are needed in order to assure safe motherhood for all.

## Introduction

Despite evidence of effective interventions to promote safe motherhood, morbidity and mortality related to pregnancy and childbirth remain major challenges to health care planners and policy makers in low- and middle-income countries. The progress towards fulfilling the fifth UN Millennium Development Goal (MDG 5), to reduce maternal mortality by 2015 by three quarters from the level of 1990, is still far off the track in most countries of the world [1]. Furthermore, countries that on a national level actually have succeeded in improving maternal health and reducing maternal mortality are still faced with big inequalities between different segments of the populations. Disadvantaged groups of women tend to have higher rates of both morbidity and mortality, and less access to safe, affordable and acceptable health care services enabling safe pregnancy and childbirth [1,2]. This “hidden” ill-health further adds to the challenge of reaching MDG 5, not only for an average but for all. Attempts have been made to reduce health inequalities between advantaged and disadvantaged populations, on global, national and sub-national levels, and ensure opportunities to all members of a society to achieve good health [3]. Most health systems are, however, inequitable, benefiting the well-off more than the disadvantaged [4] and under-utilization of health care services are typically greater where the need is biggest, in accordance with the *inverse care law *[5]. In 2009 the World Health Organization (WHO) concluded that there is still a need to better understand determinants of reproductive health in order to improve access to health services for disadvantaged groups [6].

### Maternal health in Vietnam

The proportion of people living below the poverty line in Vietnam has reduced significantly in the last decades. According to data from the Vietnam Household Living Standards Survey (VHLSS) 2006 one fifth of the population was living below the poverty line as defined by the General Statistics Office as a per capita expenditure of less than 2 559 850 VND per person and year [7]. The rate of poverty reduction has however been lower among less affluent groups [8] and ethnic minorities [9], widening gaps between different segments of the population.

Following the economic transition in the 1980s Vietnam has seen a decline in maternal mortality ratio over the past decades, from an estimated 170 in 1990 [10] to about 70 in 2009 [11]. Data on maternal mortality should however be treated with caution as no reliable official registration systems exist for maternal deaths in Vietnam, and estimates have merely been done based on survey data collected though indirect methods, such as the *sisterhood method *[12]. This is reflected by great disparities between various estimations. Analyses of existing data do however indicate considerable inequities between social groups Vietnam. According to data from 2000/2001, ethnic minority groups had a maternal mortality ratio that was almost 4 times the size of that in the ethnic majority group [12].

In 2009, UNICEF published a report on inequities in maternal and child health in Vietnam [8]. This study revealed that, although maternal health has improved over time, inequalities still existed between disadvantaged and privileged groups. Factors, such as household wealth and commune effect, were identified as important social determinants of maternal health care utilization. More recent studies have found major disparities in antenatal care utilization between urban and rural areas in Vietnam [13]. There is, however, still a need to better understand the relative importance of the different social determinants of health and potential synergy effects between them, in order to target resources efficiently and achieve MDG 5 in an equitable manner. The aim of the study is therefore to reassess available data on antenatal care coverage and skilled birth attendance in order to identify disadvantaged populations and better understand inequity in maternal health for all in Vietnam, with a special focus on ethnicity.

## Methods

### Study data

Several national surveys have been carried out in Vietnam during the last decades covering different aspects of maternal health. One of the more comprehensive ones, the Multiple Indicator Cluster Survey (MICS), has been designed by UNICEF with the aim to collect internationally comparable data on the state of women and children. Conducted in 2006, MICS 3 covered a total of 8,356 households representing the whole of Vietnam. The sampling was based on 250 census enumeration areas (EAs) representing 8 regions in Vietnam: Red river delta, North west, North east, North central coast, South central coast, Central highlands, South east and Mekong river delta. From these EAs a systematic sample of 1/3 of households were selected and eligible for inclusion (ref MICS3 final report). Women of reproductive age (15–49 years) in these households were interviewed about demographic characteristics, reproductive history, pregnancy, postnatal care, as well as immunization and nutrition. The sampling method of the survey has been reported in detail elsewhere [14]. For this study data from MICS 3 have been accessed and analyzed with authorization of UNICEF.

### Measurements

#### Conceptual framework

**Figure 1:**
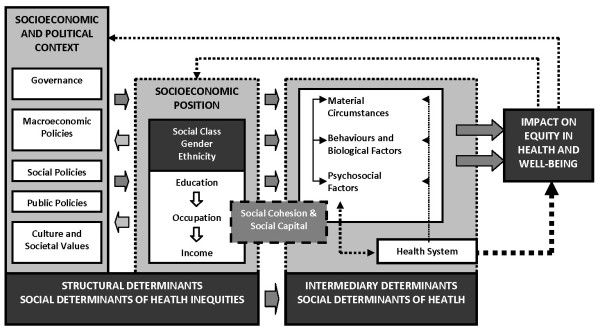
**Social determinants of health.** Figure based on a model developed by the Commission on Social Determinants of Health (CSDH). Reproduced with permission. [[15]].

The Commission on Social Determinants of Health (CSDH) set up by the WHO has developed a conceptual framework, based on previous research, with the aim to aid researchers, policy makers and health planners in their work to reduce health inequity. The framework has been summarized in Figure 1[15].

According to the CSDH framework, the factors giving rise to inequitable distribution of health and disease across social groups are termed as social determinants of health. The structural social determinants are based on social position which in turn is assigned by a complex web of societal and cultural mechanisms that generate and uphold social hierarchies. These give rise to stratification and division of power, prestige and resources and income, education and ethnicity are among the structural determinants commonly referred to. The structural determinants operate through intermediary social determinants of health. These include material circumstances, psychosocial circumstances, behavioural, and/or biological factors [15]. Improvements in public health will therefore depend not only on access to health care but on a range of other social determinants of health.

### Structural determinants

The structural determinants selected for this study are mothers’ *educationethnicity* and *household wealth*. Education is a variable frequently used in epidemiological studies. It captures many different factors determining health and wellbeing, being associated with socioeconomic position, as well as knowledge and skills affecting the ability to understand health risks and access health care [15]. In this study women who have never attended school have been compared with those who have attended at least primary school, in order to isolate the impact of schooling no matter the level.

Ethnicity is associated with social division in most contexts. People who belong to an ethnic minority tend to be marginalized and discriminated, affecting prospects of social status and opportunities. Such oppression is often associated with poor mental and physical health [15]. Vietnam is an ethnically diverse country with 54 different ethnic groups and 7 major language families. The majority of the Vietnamese population belongs to the ethnic group called Kinh. Together with people of Chinese ethnicity, they constitute the most privileged group. While the remaining ethnic minority groups account for only about 14% of the population, they make up for almost half of the poor in Vietnam [9].

Household wealth is correlated with better living conditions as well as access to services which may have a direct impact on health. It is further associated to social status and position in society. There is a reverse causality between wealth and health as income levels and expenditures may also be affected by health status [15]. No direct measurements of income or living standards were collected in MICS 3. The data do however allow for indirect estimation and the creation of a wealth index and wealth quintiles on household level by principal component analysis (PCA) [16,17]. Assets included were radio, TV, mobile phone, telephone, fridge, bike, motorbike, car, boat, electricity, type of floor, type of roof, type of wall, type of fuel, sleeping room, water and sanitation [14]. In order to isolate the risk associated with being poor, the wealth quintiles have in this study been categorized as *poor * including the bottom wealth quintile of the population, and *non-poor * the remaining four quintiles.

**Figure 2:**
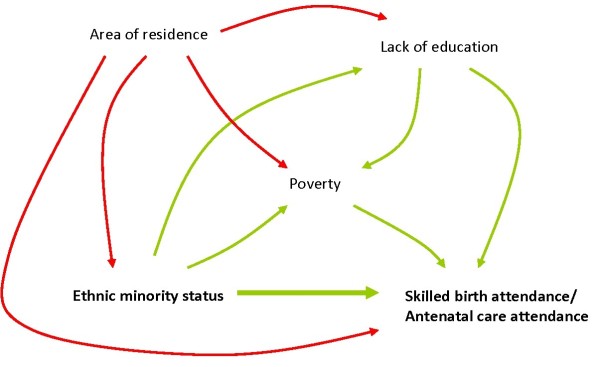
Directed Acyclic Graph (DAG) on causal effects and mediators of ethnicity and skilled birth attendance/antenatal care attendance, Vietnam 2006.

The relations between the structural determinants are described through a causal diagram, Directed Acyclic Graph (DAG), as depicted in Figure 2. The ill health of ethnic minorities is often attributed to poverty and poor education, and there is definitely a mediating effect of these variables on the association between ethnicity and the outcome [18]. However, studies have indicated effects of ethnicity over and above income and education, implying a direct effect of ethnicity in itself. In the diagram we have also included living area as a confounder even if it can be argued that place of residence is an intermediary determinant not to be mixed with the structural variables. Nevertheless, remoteness and other geographical constraints have an obvious impact not only on access to health care but also a strong association to poverty and regional cultural differences as express in ethnicity.

### Intermediary determinants

The intermediary determinants *living area, maternal age at delivery,* and *marital status* have been included in this study. Living area, as defined by rural or urban residence in the MICS3 data set, is a variable associated with the environmental and socioeconomic context, which may impact on health in many ways. Exposure to environmental risks as well as access to services tends to vary greatly between urban and rural areas, which constitute the elements of comparison in this study. Age and marital status are also associated to both wealth and social status [15].

The outcome variables in focus for this study, *antenatal care coverage* and *skilled birth attendance*, are also categorized as intermediary determinants for high level outcomes such as maternal and newborn health. In the model presented in Figure 1, these variables both fall under *health system*. Being key determinants of maternal newborn and health, they have both been selected indicators of MDG 5. Antenatal care coverage is defined as the percentage of women aged 15–49 years with a live birth during the two years preceding the survey who received antenatal care at least once by skilled health personnel. Skilled health personnel are accredited health professionals such as doctor, nurse, midwife or auxiliary midwife and do not include traditional birth attendants. Skilled birth attendance is defined as the percentage of women aged 15–49 years who with a live birth during the two years preceding the survey attended by skilled health personnel [14].

### Statistical analysis

Social determinants of health inequities are complex and interactive. Therefore it is important to assess factors both independently and in synergy [19]. When analyzing data through logistic regression it is also essential to separate structural and intermediary determinants [20]. Since the aim of this study was to identify disadvantaged groups and examine the scope of inequity, all structural determinants were considered for the analysis. Intermediary determinants were first assessed in relation to antenatal care coverage and skilled birth attendance in a bivariate logistic regression model. The intermediary determinants that were associated with the outcome variables (p < 0.05) were included in a multivariate regression model. In the next step stratifications by structural determinants were performed and multivariate regressions were conducted. To further explore not only the association but to better understand the interaction between different structural determinants and the outcome, an analysis using g-computation was performed [21]. G-computation is a method used within the field of causal inference estimation [22] and based on the causal diagram presented in Figure 2 an analysis of the natural direct effect of ethnicity on the outcome variables was calculated. Statistical analyses using the “binreg”, tabodds” and “gformula” commands in STATA 12 were performed.

## Results

### Sample characteristics

A total of 9,473 women of reproductive age (15–49 years) were interviewed in the MICS 3 survey in Vietnam, with a response rate of 94%. The 1,023 interviewed women who had given birth to a live child two years preceding the survey were included. Of these women 78% (797/1,023) lived in rural areas, and 28% (284/1,023) in households classified as poor. Almost three quarters (71%) belonged to one of the two ethnic majority groups, Kinh and Chinese, and 87% had attended school. Only 7% were below the age of 20 years by the time of the interview.

### Antenatal care coverage

Data on antenatal care had been successfully collected for 1,016 women. Analyses reveal that 87% (883/1016) had received antenatal care by skilled personnel at least once during their last completed pregnancy (Table 1). Controlling for living area, multivariate analyses show that education, wealth and ethnicity were all significantly associated with antenatal care coverage. The greatest discrepancy was found between ethnic groups, where the ethnic minority women had a more than threefold risk of not receiving antenatal care (OR 3.5, 95% CI 1.95–6.35) compared to women belongning to the ethnic majority group.

**Table 1:** Multivariate analysis of antenatal care coverage and selected structural determinants (adjusted for living area), percentage and adjusted odds ratio (OR), women age 15–49, MICS 3 Vietnam 2006 (n = 1,016)

	**Attended antenatal care (n)**	**No antenatal care (n)**	**OR (95% CI)**
**Education**			
Educated	826	75	Ref
Uneducated	57	58	2.78 (1.68-4.60)**
**Ethnicity**			
Kinh/Chinese	692	30	Ref
Other	191	103	3.51 (1.95–6.35)**
**Wealth**			
Non-poor	700	34	Ref
Poor	183	99	2.62 (1.46–4.67)*

*p < 0.05 **p < 0.001.

Stratified logistic regressions reveal an increased risk for ethnic minority women of not utilizing antenatal care, independent of economic status (Table 2). The odds ratio (OR) for poor ethnic minority women was 3.06 (95% CI 1.27–7.41) and 4.27 (95% CI 1.81–10.09) for non-poor ethnic minority women, compared to ethnic majority women of the same wealth status. Wealth was equally associated with the outcome independent of ethnicity. Women who belonged to an ethnic minority and were living in a poor household had an almost 10 folded risk (OR 9.69, 95% CI 5.15–18.24) of not receiving antenatal care, as compared to ethnic majority women living in a non-poor household.

**Table 2:** Multivariate analysis of antenatal care coverage and wealth stratified by ethnicity (adjusted for living area and education), women age 15–49, MICS 3 Vietnam 2006 (n = 1,016)

		**Attended antenatal care (n)**	**No antenatal care (n)**	**OR (95% CI)**	**OR (95% CI)**	**OR (95% CI)**
Kinh/Chinese	Non-poor	639	23	Ref	0.31 (0.12–0.82)*	0.23 (0.10–0.55)**
	Poor	53	7	3.21 (1.22–8.41)*	Ref	0.76 (0.27–2.17)
Other	Non-poor	61	11	4.27 (1.81–10.09)**	1.32 (0.46–3.76)	Ref
	Poor	130	92	9.69 (5.15–18.24)**	3.06 (1.27–7.41)*	2.37 (1.13–4.96)*

*p < 0.05 **p < 0.001.

When stratifying the education variable by ethnicity it was found that being uneducated was associated with an increased risk of not receiving antenatal care in the ethnic minority group, with an OR of 2.95 (95% CI 1.70–5.12). No such relation could be established within the ethnic majority group. For educated women, belonging to the ethnic minority group was found to be a risk factor, but not for uneducated women. The OR of not receiving antenatal care was 3.30 (95% CI 1.72–6.35) for educated ethnic minority women as compared to educated ethnic majority women.

### Skilled birth attendance

Out of the 1,023 women of reproductive age with a live birth during the last two years, data on birth attendance had been recorded for 1,021. In 2006, 82% of the women had been attended by skilled personnel (Table 3). Multivariate analyses of determinants show that education, wealth, and ethnicity were all significantly associated with skilled birth attendance. After controlling for significant intermediary and structural determinants, the greatest discrepancy was found between ethnic groups, where the risk of not receiving skilled birth attendance was almost 7 times higher (OR 6.85, 95% CI 4.01–11.7) in the ethnic minority group, as compared to the Kinh/Chinese group.

**Table 3:** Multivariate analysis of skilled birth attendance and selected structural determinants (adjusted for age and living area), percentage and adjusted odds ratios (OR), women age 15–49 years, MICS 3 Vietnam 2006 (n = 1,021)

	**Received skilled birth attendance (n)**	**No skilled birth attendance (n)**	**OR (95% CI)**
**Education**			
Educated	799	106	Ref
Uneducated	33	83	3.52 (2.07–6.00)**
**Ethnicity**			
Kinh/Chinese	695	31	Ref
Other	137	158	6.85 (4.01–11.7)**
**Wealth**			
Non-poor	698	41	Ref
Poor	134	148	3.20 (1.91–5.38)**

*p < 0.05 **p < 0.001.

In a stratified analysis, ethnicity was found to be associated with skilled birth attendance, independently of wealth status (Table 4). Poor women in the ethnic minority group ran a 6.27 (95% CI 2.37–16.6) times greater risk of not being attended by skilled personnel as compared to poor women in the ethnic majority group. An even greater disparity was found when comparing non-poor women in the different ethnic groupings, where ethnic minority women had an OR of 7.67 (95% CI 3.54–16.6) of not receiving skilled attendance at birth. At greatest risk of not receiving skilled birth attendance was poor women from ethnic minority groups who had an OR of 25.5 (95% CI 11.4–56.8), compared to non-poor Kinh/Chinese women.

**Table 4:** Multivariate analysis of skilled birth attendance and wealth stratified by ethnicity (adjusted for living area, age and education), women age 15–49, MICS 3 Vietnam 2006 (n = 1,021)

		**Received skilled birth attendance (n)**	**No skilled birth attendance (n)**	**OR (95% CI)**	**OR (95% CI)**	**OR (95% CI)**
Kinh/Chinese	Non-poor	645	21	Ref	0.15 (0.06–0.43)**	0.13 (0.06–0.28)**
	Poor	50	10	6.41 (2.29–18.0)**	Ref	0.63 (0.23–1.70)
Other	Non-poor	53	20	7.67 (3.54–16.6)**	1.58 (0.59–4.26)	Ref
	Poor	84	138	25.5 (11.4–56.8)**	6.27 (2.37–16.6)**	2.91 (1.42–5.99)*

*p < 0.05 **p < 0.001.

Ethnicity was further found to be a significant determinant among educated women, who ran a six folded risk (OR 6.33, 95% CI 3.33–12.0) of giving birth without skilled attendance if they belong to an ethnic minority group, as compared to educated women in the ethnic majority group. Due to small sample size, no similar associations could be established among uneducated women.

### The effects of ethnicity

The Total Causal Effect (TCE) describes the sum of all possible pathways in the DAG (Figure 2), whereas the Natural Direct Effect (NDE) describes all pathways not going through household economic status (Table 5). Thus, through g-computation we found that a major part of the effect of ethnic status on the outcome variables was mediated through household economic status. However, 25% (NDE 0.0312805/TCE 0.1231672) of the causal effect of ethnicity on skilled birth attendance and 18% (NDE 0.0156403/TCE 0.086999) on antenatal care attendance were not due to poverty (Table 5).

**Table 5:** G-computation based on causal diagram calculating the Total Causal Effect (TCE), Natural Direct Effect (NDE) and Natural Indirect Effect (NIE) of ethnicity on antenatal care and skilled birth attendance based on MICS3 data, Vietnam 2006

**Antenatal care attendance**						
	G-computation estimate	Bootstrap Std. Error	Z	P > |z|	Normal-based (95% CI)	
TCE	0.086999	0.0152105	5.72	<0.001	0.057187	0.116811
NDE	0.0156403	0.0113028	1.38	0.166	−0.0065129	0.0377934
NIE	0.0713587	0.0153506	4.65	<0.001	0.0412821	0.1014454
**Skilled birth attendance**						
	G-computation estimate	Bootstrap Std. Error	Z	P > |z|	Normal-based (95% CI)	
TCE	0.1231672	0.01671	7.37	<0.001	0.0904162	0.1559181
NDE	0.0312805	0.0129089	2.42	0.015	0.0059796	0.0565815
NIE	0.0918866	0.0124791	2.11	0.034	0.0019344	0.0508516

## Discussion

In this study we have analyzed associations between antenatal care and skilled birth attendants, and different structural determinants of health. We have also tried to capture the causal effect of ethnicity on the outcome variables through novel statistical methods. Findings show that maternal health care utilization was still, in 2006, highly inequitable in Vietnam, determined by ethnicity, education, and wealth. The biggest inequities detected in this study were related to skilled birth attendance. While practically all Kinh/Chinese women had skilled attended deliveries, less than half of ethnic minority women had the same. These findings correspond to the results of previous analysis made on national survey data from 2001–2002 [23].

The results demonstrate that ethnicity is an important social determinant for maternal health care utilization in Vietnam, and that ethnic minority women form a clearly disadvantaged group. Ethnic minorities tend to reside in rural areas and poor households, as well as have low education [9,24]. Physical distances to health facilities as well as lack of transportation and means to accommodate women and their family members, are factors previously identified as explaining the lower rates of maternal health care utilization in areas inhabited mainly by ethnic minority groups [25,26]. Studies have shown that as many as half of all women in remote and mountainous areas in Vietnam deliver their babies at home [27]. Shortage of equipment, drugs and staff in the health facilities [28], and indirect costs associated with seeking care are other factors of importance [25]. Previous research reveals a relationship between use and quality of antenatal care and giving birth in health facilities [29].

In an analysis of ethnicity and social development in Vietnam published by the World Bank in 2009, the reasons for ethnic minority groups being disadvantaged are suggested to be multiple and interacting. The findings can be summarized as ethnic minorities having fewer physical assets, as well as social assets (such as education and access to services), residing in remote areas, and not benefitting from government poverty reduction programs. Added to this may be other socio-cultural factors excluding them from economic development [9]. The results indicate that as much as a quarter of the causal effect of ethnicity on lack of skilled birth attendance is mediated through other factors than being an effect of poverty. The use of statistical methods like G-computation allows us to look at the data from different angles and a better understanding is achieved through the use of various statistical methods complementing each other. The results of the G-computation verifies the results from the logistic regression models that ethnicity is not solely a matter of economy. At the same time it also reinforces the fact that ethnic minorities are marginalized and impoverished, showing that large improvements could be made in the provision of maternal health services if poverty among these groups was alleviated.

This study shows that the factors above cannot solely explain the health inequities observed between ethnic groups. Belonging to en ethnic minority is a risk factor in itself working in synergy with other factors. Although we can expect an overlapping effect between the structural determinants analyzed, those most at risk of not utilizing maternal health care are women of ethnic minorities who lack education and live in poor households.

Several studies, mainly of qualitative design, have made attempts to explain why ethnic minority groups are disadvantaged in terms of health care in Vietnam. Among barriers identified for utilizing health care services are traditional practices such as home deliveries [24-26,30] and complex rituals surrounding births [24]. Perceived negative attitudes from health care personnel, language barriers [24] and feelings of disempowerment and voiceless [9] are also mentioned. Several studies have found gender sensitivity to be of particular importance to some ethnic minority groups, as many women do not feel comfortable being cared for by male personnel [24-26].

It should be stressed that ethnic minority women in Vietnam make up a highly heterogenic group, of various ethnicities with differences in culture, language, place of residence, economic status and education levels [9]. To fully understand these differences and their relation to the social determinants of maternal health care utilization is of outermost importance for targeting scarce resources efficiently. Empowering ethnic minority groups and involving them in the planning and implementation of interventions through open dialogues have shown to be successful elsewhere [31-33].

The fact that the data used in this study was collected in 2006 is of concern. One can assume that much has happened since and that conclusions drawn from these data may be outdated. Collecting information through recall of reproductive history generates data that is even older than the date of collection, and may also be associated with a risk of recall bias. The broad patterns of social determinants of health care utilization are however likely to remain, which is supported by findings made by Boerma et al. in 2008. Analyzing survey data from 54 countries on maternal and newborn health interventions, they found that patterns of inequity tend to be consistent over time [34].

To fully grasp the current situation for disadvantaged groups in Vietnam updated data is however of essence and the collection of reliable information on incidence and causes of maternal mortality, through strengthening of civil registration systems, will be of vital importance to enable promotion of maternal health and survival.

### Concluding remarks and recommendations

According to findings made in this study, ethnic minority women constitute a disadvantaged group in Vietnam. The fact that they tend to reside in rural areas, lack education and live in poor households only partly explains their increased risk of not receiving vital maternal health care. This study shows that belonging to an ethnic minority group is a risk in itself. The complex interactions and synergy effects between different social determinants of health highlighted in this study stresses the importance of parallel investments in other sectors than health care, such as education, income generating activities, women empowerment, and infrastructure, in order to promote safe motherhood.

## Abbreviations

CI: Confidence interval; CSDH: Commission of social determinants of health; DAG: Directed acyclic graph; EA: Enumeration areas; MDG: Millennium development goal; MICS: Multiple Indicator cluster survey; NDE: Natural direct effect; NIE: Natural Indirect effect; OR: Odds ratio; PCA: Principal component analysis; TCE: Total causal effect; VHLSS: Vietnam household living standards survey; WHO: World Health Organization.

## Competing interests

The authors declare no competing interest in relation to the article.

## Author’s contributions

EG and MM planned the study and performed statistical analyses. EG prepared the first draft of the manuscript. DPH and MM provided professional comments and edited the final manuscript. All authors read and approved the final manuscript.

## References

[B1] The Millennium Development Goals Report 2011 2011 United Nations: New York

[B2] Irwin A Valentine N Brown C Loewenson R Solar O Brown H Koller T Vega J The commission on social determinants of health: tackling the social roots of health inequities PLoS Med 2006 3 e106 10.1371/journal.pmed.0030106 16681414 PMC1459479

[B3] Culyer AJ Equity - some theory and its policy implications J Med Ethics 2001 27 275 283 10.1136/jme.27.4.275 11479360 PMC1733434

[B4] Gwatkin DR Bhuiya A Victora CG Making health systems more equitable Lancet 2004 364 1273 1280 10.1016/S0140-6736(04)17145-6 15464189

[B5] Hart JT The inverse care law Lancet 1971 1 405 412 4100731 10.1016/s0140-6736(71)92410-x

[B6] Global Forum for Health Research World Health Organization: Perceived research priorities in sexual and reproductive health for low- and middle-income countries: results from a survey 2009 Geneva, Switzerland: Global Forum for Health Research

[B7] Baulch B Dat VH Poverty dynamics in Vietnam, 2002–2006. Background paper for the 2008–2009 Vietnam Poverty Assessment 2008

[B8] Knowles JC Bales S Cuong LQ Oanh TTM Luon DH Health Equity in Viet Nam: a situation analysis focused on maternal and child mortality. Background paper prepared for the UNICEF Consultancy on Equity in Access to Quality Healthcare for Women and Children, April 8–10, 2009 2009 UNICEF: Ha Long, Viet Nam

[B9] Country Social Analysis Ethnicity and social development in Vietnam. Summary report 2009 Washington, D.C: The World Bank

[B10] Trends in Maternal Mortality 1990–2008 2010 Geneva: World Health Organization

[B11] The Vietnam Population and Housing Census Major Findings 2010 Hanoi: Vietnam General Statistics Office

[B12] Maternal Mortality in Vietnam 2000–2001: an in-depth analysis of causes and determinants Maternal Mortality in Vietnam 2000–2001: an in-depth analysis of causes and determinants 2005 Hanoi: World Health Organization

[B13] Tran TK Nguyen CT Nguyen HD Eriksson B Bondjers G Gottvall K Ascher H Petzold M Urban - rural disparities in antenatal care utilization: a study of two cohorts of pregnant women in Vietnam BMC Health Serv Res 2011 11 120 10.1186/1472-6963-11-120 21605446 PMC3224373

[B14] Monitoring the Situation of Children and Women - Viet Nam Multiple Indicator Cluster Survey Final Report 2007 Hanoi: General Statistics Office, UNICEF

[B15] Commission on Social Determinants of Health A Conceptual Framework for Action on the Social Determinants of Health 2007 Geneva: World Health Organization

[B16] Filmer D Pritchett LH Estimating wealth effects without expenditure data–or tears: an application to educational enrollments in states of India Demography 2001 38 115 132 11227840 10.1353/dem.2001.0003

[B17] Ruthstein SO Johnson K The DHS Wealth Index. In DHS Comparative Report no 6 2004 Maryland: ORC Macro, Calverton

[B18] Salway S Platt L Chowbey P Harriss K Bayliss E Long-term illhealth, poverty and ethnicity 2007 Plymouth: Joseph Rowntree Foundation. The Policy Press

[B19] Wirth ME Balk D Delamonica E Storeygard A Sacks E Minujin A Setting the stage for equity-sensitive monitoring of the maternal and child health Millennium Development Goals Bull World Health Organ 2006 84 519 527 10.2471/BLT.04.019984 16878225 PMC2627391

[B20] Victora CG Huttly SR Fuchs SC Olinto MT The role of conceptual frameworks in epidemiological analysis: a hierarchical approach Int J Epidemiol 1997 26 224 227 10.1093/ije/26.1.224 9126524

[B21] Neugebauer R van der Laan M G-computation estimation for causal inference with complex longitudinal data Comput Stat Data Anal 2006 51 1676 1697 10.1016/j.csda.2006.06.016

[B22] Snowden JM Rose S Mortimer KM Implementation of G-computation on a simulated data set: demonstration of a causal inference technique Am J Epidemiol 2011 173 731 738 10.1093/aje/kwq472 21415029 PMC3105284

[B23] Sepehri A Sarma S Simpson W Moshiri S How important are individual, household and commune characteristics in explaining utilization of maternal health services in Vietnam? Soc Sci Med 2008 67 1009 1017 10.1016/j.socscimed.2008.06.005 18635302

[B24] Knowledge and Behaviour of Ethnic Minorities on Reproductive Health 2007 Hanoi: UNFPA

[B25] Reproductive Health of H’mong People in Ha Giang Province Medical Anthropology Perspective 2008 Hanoi: UNFPA

[B26] Childbirth in Ethnic Minority Communities A qualitative Study in Binh Dinh Province 2008 Hanoi: UNFPA

[B27] Duong DV Binns CW Lee AH Utilization of delivery services at the primary health care level in rural Vietnam Soc Sci Med 2004 59 2585 2595 10.1016/j.socscimed.2004.04.007 15474211

[B28] Graner S Mogren I le Duong Q Krantz G Klingberg-Allvin M Maternal health care professionals’ perspectives on the provision and use of antenatal and delivery care: a qualitative descriptive study in rural Vietnam BMC Public Health 2010 10 608 10.1186/1471-2458-10-608 20946681 PMC3091560

[B29] Trinh LT Dibley MJ Byles J Determinants of antenatal care utilization in three rural areas of Vietnam Public Health Nurs 2007 24 300 310 10.1111/j.1525-1446.2007.00638.x 17553019

[B30] Van Vo T Hoat LN Jan van Schie T Situation of the Kinh poor and minority women and their use of the Maternal Care and Family Planning Service in Nam Dong Mountainous District, Thuathien-Hue Province Vietnam. Rural Remote Health 2004 4 255 15887985

[B31] Wallin L Malqvist M Nga NT Eriksson L Persson LA Hoa DP Huy TQ Duc DM Ewald U Implementing knowledge into practice for improved neonatal survival; a cluster-randomised, community-based trial in Quang Ninh province Vietnam. BMC Health Serv Res 2011 11 239 10.1186/1472-6963-11-239 PMC3192673 21951770

[B32] Turan JM Tesfagiorghis M Polan ML Evaluation of a community intervention for promotion of safe motherhood in Eritrea J Midwifery Womens Health 2011 56 8 17 10.1111/j.1542-2011.2010.00001.x 21323845 PMC3498940

[B33] Renju JR Andrew B Medard L Kishamawe C Kimaryo M Changalucha J Obasi A Scaling up adolescent sexual and reproductive health interventions through existing government systems? A detailed process evaluation of a school-based intervention in Mwanza region in the northwest of Tanzania J Adolesc Health 2011 48 79 86 10.1016/j.jadohealth.2010.05.007 21185528

[B34] Boerma JT Bryce J Kinfu Y Axelson H Victora CG Mind the gap: equity and trends in coverage of maternal, newborn, and child health services in 54 Countdown countries Lancet 2008 371 1259 1267 18406860 10.1016/S0140-6736(08)60560-7

